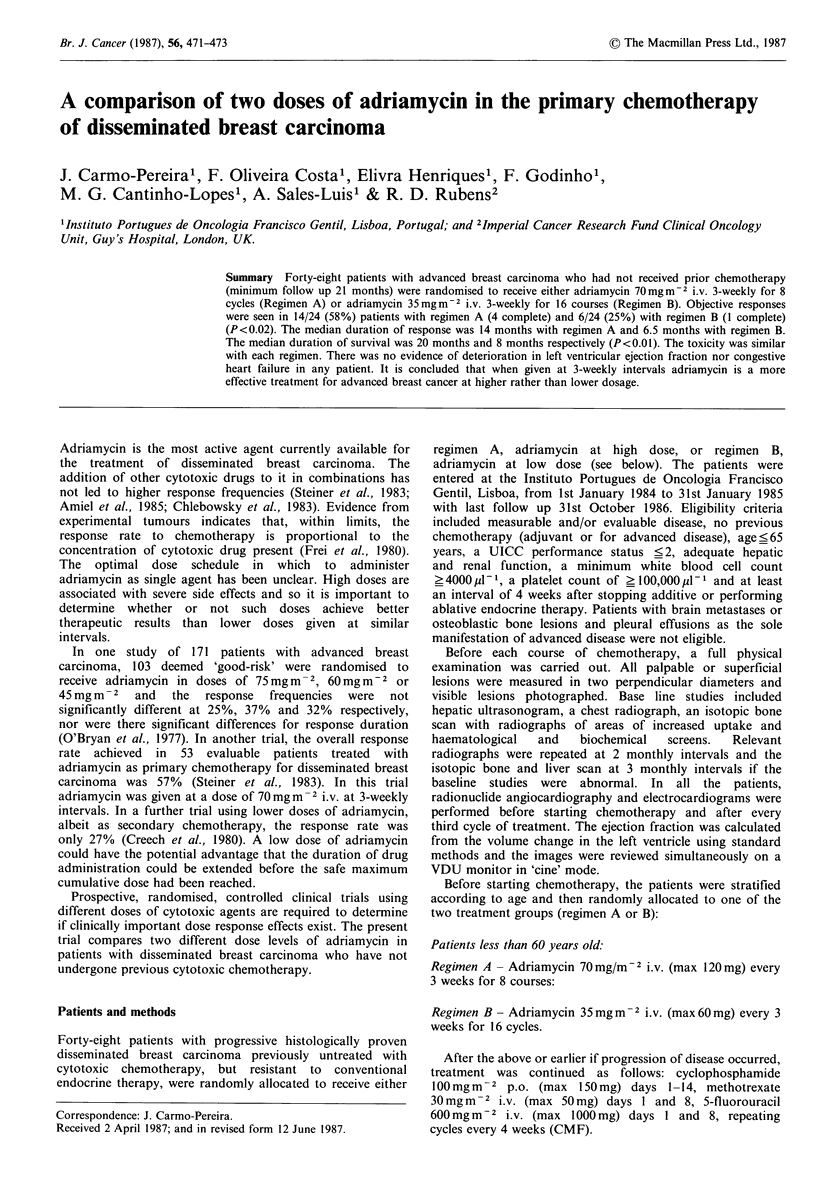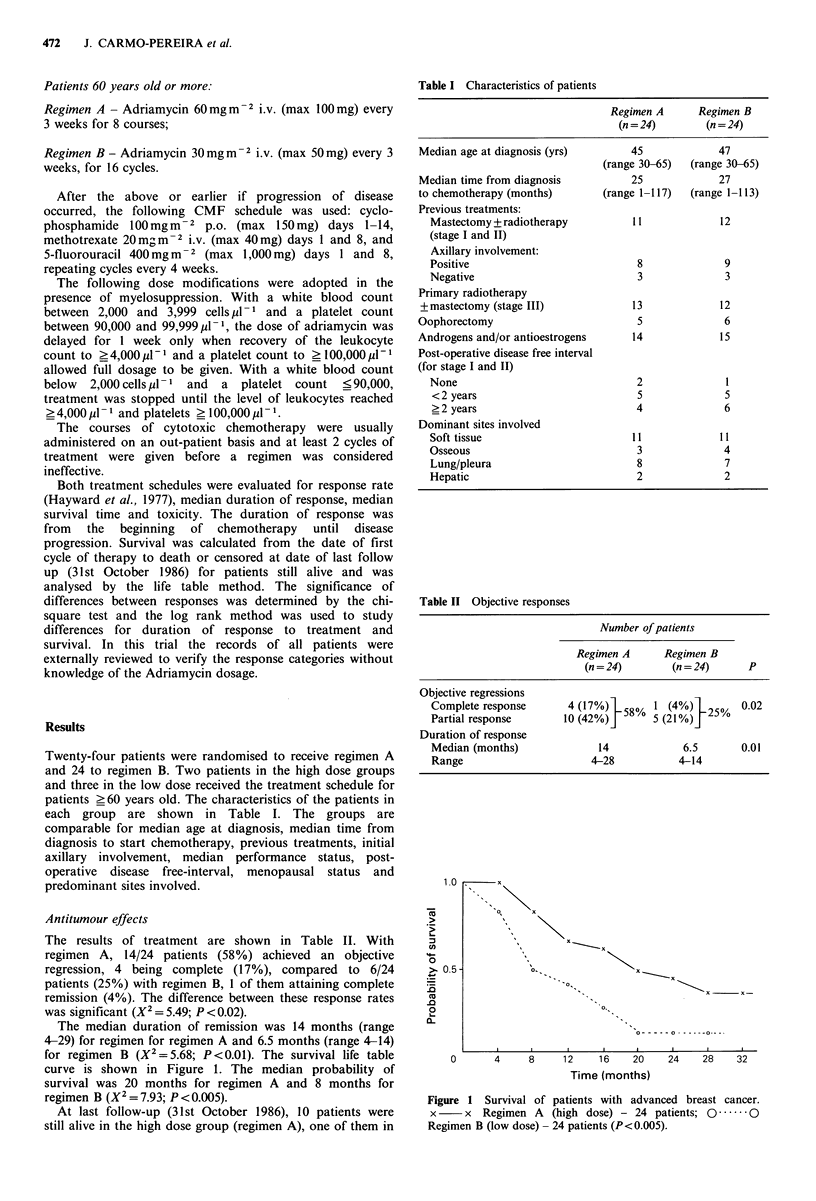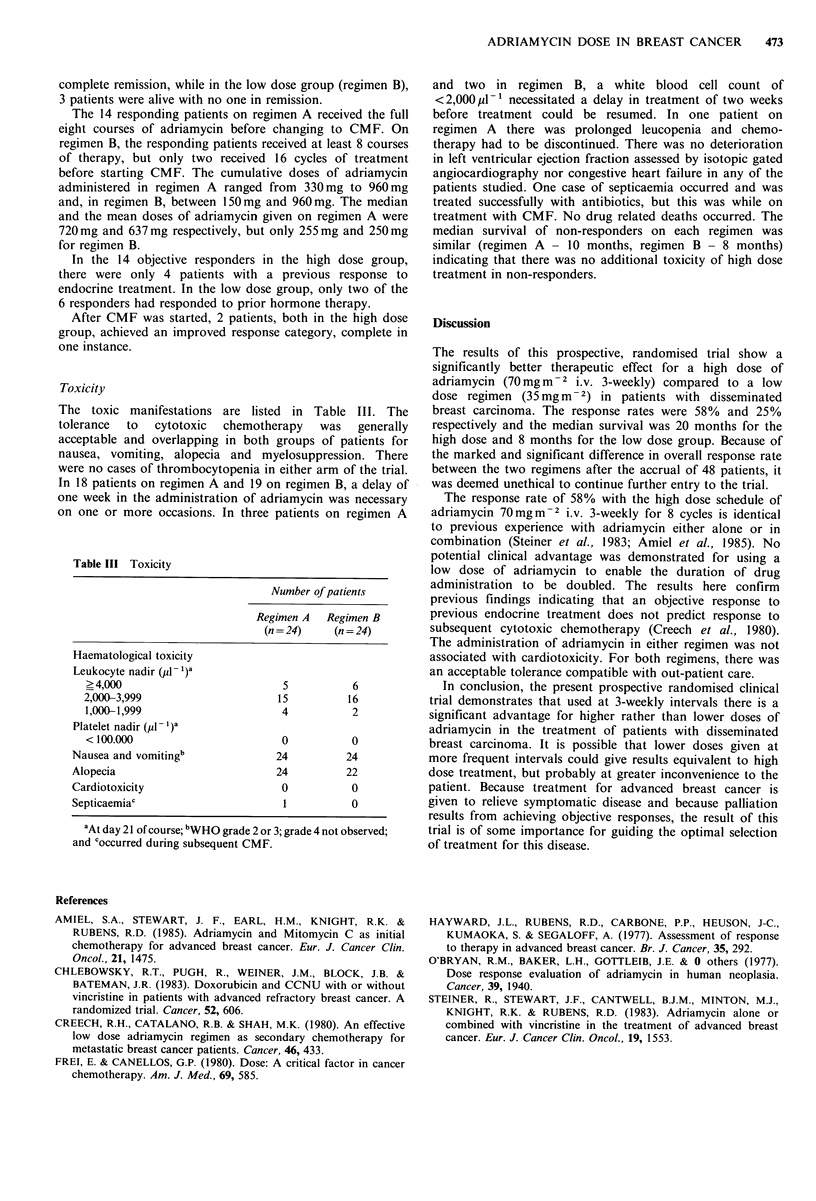# A comparison of two doses of adriamycin in the primary chemotherapy of disseminated breast carcinoma.

**DOI:** 10.1038/bjc.1987.226

**Published:** 1987-10

**Authors:** J. Carmo-Pereira, F. O. Costa, E. Henriques, F. Godinho, M. G. Cantinho-Lopes, A. Sales-Luis, R. D. Rubens

**Affiliations:** Instituto Portugues de Oncologia Francisco Gentil, Lisboa, Portugal.

## Abstract

Forty-eight patients with advanced breast carcinoma who had not received prior chemotherapy (minimum follow up 21 months) were randomised to receive either adriamycin 70 mg m-2 i.v. 3-weekly for 8 cycles (Regimen A) or adriamycin 35 mg m-2 i.v. 3-weekly for 16 courses (Regimen B). Objective responses were seen in 14/24 (58%) patients with regimen A (4 complete) and 6/24 (25%) with regimen B (1 complete) (P less than 0.02). The median duration of response was 14 months with regimen A and 6.5 months with regimen B. The median duration of survival was 20 months and 8 months respectively (P less than 0.01). The toxicity was similar with each regimen. There was no evidence of deterioration in left ventricular ejection fraction nor congestive heart failure in any patient. It is concluded that when given at 3-weekly intervals adriamycin is a more effective treatment for advanced breast cancer at higher rather than lower dosage.


*Br. J. Cancer* (1987), **56**, 471–473 © The Macmillan Press Ltd., 1987

### A comparison of two doses of adriamycin in the primary chemotherapy of disseminated breast carcinoma

J. Carmo-Pereira[1], F. Oliveira Costa[1], Elivra Henriques[1], F. Godinho[1], M. G. Cantinho-Lopes[1], A. Sales-Luis[1] & R. D. Rubens[2]

[1]*Instituto Portugues de Oncologia Francisco Gentil, Lisboa, Portugal; and* [2]*Imperial Cancer Research Fund Clinical Oncology Unit, Guy's Hospital, London, UK.*

**Summary** Forty-eight patients with advanced breast carcinoma who had not received prior chemotherapy (minimum follow up 21 months) were randomised to receive either adriamycin 70 mg m$^{-2}$ i.v. 3-weekly for 8 cycles (Regimen A) or adriamycin 35 mg m$^{-2}$ i.v. 3-weekly for 16 courses (Regimen B). Objective responses were seen in 14/24 (58%) patients with regimen A (4 complete) and 6/24 (25%) with regimen B (1 complete) ($P<0.02$). The median duration of response was 14 months with regimen A and 6.5 months with regimen B. The median duration of survival was 20 months and 8 months respectively ($P<0.01$). The toxicity was similar with each regimen. There was no evidence of deterioration in left ventricular ejection fraction nor congestive heart failure in any patient. It is concluded that when given at 3-weekly intervals adriamycin is a more effective treatment for advanced breast cancer at higher rather than lower dosage.

Adriamycin is the most active agent currently available for the treatment of disseminated breast carcinoma. The addition of other cytotoxic drugs to it in combinations has not led to higher response frequencies (Steiner *et al.*, 1983; Amiel *et al.*, 1985; Chlebowsky *et al.*, 1983). Evidence from experimental tumours indicates that, within limits, the response rate to chemotherapy is proportional to the concentration of cytotoxic drug present (Frei *et al.*, 1980). The optimal dose schedule in which to administer adriamycin as single agent has been unclear. High doses are associated with severe side effects and so it is important to determine whether or not such doses achieve better therapeutic results than lower doses given at similar intervals.

In one study of 171 patients with advanced breast carcinoma, 103 deemed 'good-risk' were randomised to receive adriamycin in doses of 75 mg m$^{-2}$, 60 mg m$^{-2}$ or 45 mg m$^{-2}$ and the response frequencies were not significantly different at 25%, 37% and 32% respectively, nor were there significant differences for response duration (O'Bryan *et al.*, 1977). In another trial, the overall response rate achieved in 53 evaluable patients treated with adriamycin as primary chemotherapy for disseminated breast carcinoma was 57% (Steiner *et al.*, 1983). In this trial adriamycin was given at a dose of 70 mg m$^{-2}$ i.v. at 3-weekly intervals. In a further trial using lower doses of adriamycin, albeit as secondary chemotherapy, the response rate was only 27% (Creech *et al.*, 1980). A low dose of adriamycin could have the potential advantage that the duration of drug administration could be extended before the safe maximum cumulative dose had been reached.

Prospective, randomised, controlled clinical trials using different doses of cytotoxic agents are required to determine if clinically important dose response effects exist. The present trial compares two different dose levels of adriamycin in patients with disseminated breast carcinoma who have not undergone previous cytotoxic chemotherapy.

### Patients and methods

Forty-eight patients with progressive histologically proven disseminated breast carcinoma previously untreated with cytotoxic chemotherapy, but resistant to conventional endocrine therapy, were randomly allocated to receive either regimen A, adriamycin at high dose, or regimen B, adriamycin at low dose (see below). The patients were entered at the Instituto Portugues de Oncologia Francisco Gentil, Lisboa, from 1st January 1984 to 31st January 1985 with last follow up 31st October 1986. Eligibility criteria included measurable and/or evaluable disease, no previous chemotherapy (adjuvant or for advanced disease), age $\leq 65$ years, a UICC performance status $\leq 2$, adequate hepatic and renal function, a minimum white blood cell count $\geq 4000\,\mu l^{-1}$, a platelet count of $\geq 100{,}000\,\mu l^{-1}$ and at least an interval of 4 weeks after stopping additive or performing ablative endocrine therapy. Patients with brain metastases or osteoblastic bone lesions and pleural effusions as the sole manifestation of advanced disease were not eligible.

Before each course of chemotherapy, a full physical examination was carried out. All palpable or superficial lesions were measured in two perpendicular diameters and visible lesions photographed. Base line studies included hepatic ultrasonogram, a chest radiograph, an isotopic bone scan with radiographs of areas of increased uptake and haematological and biochemical screens. Relevant radiographs were repeated at 2 monthly intervals and the isotopic bone and liver scan at 3 monthly intervals if the baseline studies were abnormal. In all the patients, radionuclide angiocardiography and electrocardiograms were performed before starting chemotherapy and after every third cycle of treatment. The ejection fraction was calculated from the volume change in the left ventricle using standard methods and the images were reviewed simultaneously on a VDU monitor in 'cine' mode.

Before starting chemotherapy, the patients were stratified according to age and then randomly allocated to one of the two treatment groups (regimen A or B):

*Patients less than 60 years old:*

*Regimen A* – Adriamycin 70 mg/m$^{-2}$ i.v. (max 120 mg) every 3 weeks for 8 courses:

*Regimen B* – Adriamycin 35 mg m$^{-2}$ i.v. (max 60 mg) every 3 weeks for 16 cycles.

After the above or earlier if progression of disease occurred, treatment was continued as follows: cyclophosphamide 100 mg m$^{-2}$ p.o. (max 150 mg) days 1–14, methotrexate 30 mg m$^{-2}$ i.v. (max 50 mg) days 1 and 8, 5-fluorouracil 600 mg m$^{-2}$ i.v. (max 1000 mg) days 1 and 8, repeating cycles every 4 weeks (CMF).

Correspondence: J. Carmo-Pereira.
Received 2 April 1987; and in revised form 12 June 1987.

*Patients 60 years old or more:*

*Regimen A* – Adriamycin $60\,mg\,m^{-2}$ i.v. (max 100 mg) every 3 weeks for 8 courses;

*Regimen B* – Adriamycin $30\,mg\,m^{-2}$ i.v. (max 50 mg) every 3 weeks, for 16 cycles.

After the above or earlier if progression of disease occurred, the following CMF schedule was used: cyclophosphamide $100\,mg\,m^{-2}$ p.o. (max 150 mg) days 1–14, methotrexate $20\,mg\,m^{-2}$ i.v. (max 40 mg) days 1 and 8, and 5-fluorouracil $400\,mg\,m^{-2}$ (max 1,000 mg) days 1 and 8, repeating cycles every 4 weeks.

The following dose modifications were adopted in the presence of myelosuppression. With a white blood count between 2,000 and 3,999 cells $\mu l^{-1}$ and a platelet count between 90,000 and 99,999 $\mu l^{-1}$, the dose of adriamycin was delayed for 1 week only when recovery of the leukocyte count to $\geqq 4{,}000\,\mu l^{-1}$ and a platelet count to $\geqq 100{,}000\,\mu l^{-1}$ allowed full dosage to be given. With a white blood count below 2,000 cells $\mu l^{-1}$ and a platelet count $\leqq 90{,}000$, treatment was stopped until the level of leukocytes reached $\geqq 4{,}000\,\mu l^{-1}$ and platelets $\geqq 100{,}000\,\mu l^{-1}$.

The courses of cytotoxic chemotherapy were usually administered on an out-patient basis and at least 2 cycles of treatment were given before a regimen was considered ineffective.

Both treatment schedules were evaluated for response rate (Hayward *et al.*, 1977), median duration of response, median survival time and toxicity. The duration of response was from the beginning of chemotherapy until disease progression. Survival was calculated from the date of first cycle of therapy to death or censored at date of last follow up (31st October 1986) for patients still alive and was analysed by the life table method. The significance of differences between responses was determined by the chi-square test and the log rank method was used to study differences for duration of response to treatment and survival. In this trial the records of all patients were externally reviewed to verify the response categories without knowledge of the Adriamycin dosage.

### Results

Twenty-four patients were randomised to receive regimen A and 24 to regimen B. Two patients in the high dose groups and three in the low dose received the treatment schedule for patients $\geqq 60$ years old. The characteristics of the patients in each group are shown in Table I. The groups are comparable for median age at diagnosis, median time from diagnosis to start chemotherapy, previous treatments, initial axillary involvement, median performance status, post-operative disease free-interval, menopausal status and predominant sites involved.

*Antitumour effects*

The results of treatment are shown in Table II. With regimen A, 14/24 patients (58%) achieved an objective regression, 4 being complete (17%), compared to 6/24 patients (25%) with regimen B, 1 of them attaining complete remission (4%). The difference between these response rates was significant ($X^2 = 5.49$; $P < 0.02$).

The median duration of remission was 14 months (range 4–29) for regimen for regimen A and 6.5 months (range 4–14) for regimen B ($X^2 = 5.68$; $P < 0.01$). The survival life table curve is shown in Figure 1. The median probability of survival was 20 months for regimen A and 8 months for regimen B ($X^2 = 7.93$; $P < 0.005$).

At last follow-up (31st October 1986), 10 patients were still alive in the high dose group (regimen A), one of them in

**Table I** Characteristics of patients

| | *Regimen A* (*n* = *24*) | *Regimen B* (*n* = *24*) |
|---|---|---|
| Median age at diagnosis (yrs) | 45 (range 30–65) | 47 (range 30–65) |
| Median time from diagnosis to chemotherapy (months) | 25 (range 1–117) | 27 (range 1–113) |
| Previous treatments: | | |
| Mastectomy ± radiotherapy (stage I and II) | 11 | 12 |
| Axillary involvement: | | |
| Positive | 8 | 9 |
| Negative | 3 | 3 |
| Primary radiotherapy ± mastectomy (stage III) | 13 | 12 |
| Oophorectomy | 5 | 6 |
| Androgens and/or antioestrogens | 14 | 15 |
| Post-operative disease free interval (for stage I and II) | | |
| None | 2 | 1 |
| <2 years | 5 | 5 |
| ≧2 years | 4 | 6 |
| Dominant sites involved | | |
| Soft tissue | 11 | 11 |
| Osseous | 3 | 4 |
| Lung/pleura | 8 | 7 |
| Hepatic | 2 | 2 |

**Table II** Objective responses

| | *Number of patients* | | |
|---|---|---|---|
| | *Regimen A* (*n* = *24*) | *Regimen B* (*n* = *24*) | *P* |
| Objective regressions | | | |
| Complete response | 4 (17%) } 58% | 1 (4%) } 25% | 0.02 |
| Partial response | 10 (42%) | 5 (21%) | |
| Duration of response | | | |
| Median (months) | 14 | 6.5 | 0.01 |
| Range | 4–28 | 4–14 | |

**Figure 1** Survival of patients with advanced breast cancer. x——x Regimen A (high dose) – 24 patients; O······O Regimen B (low dose) – 24 patients ($P < 0.005$).

complete remission, while in the low dose group (regimen B), 3 patients were alive with no one in remission.

The 14 responding patients on regimen A received the full eight courses of adriamycin before changing to CMF. On regimen B, the responding patients received at least 8 courses of therapy, but only two received 16 cycles of treatment before starting CMF. The cumulative doses of adriamycin administered in regimen A ranged from 330 mg to 960 mg and, in regimen B, between 150 mg and 960 mg. The median and the mean doses of adriamycin given on regimen A were 720 mg and 637 mg respectively, but only 255 mg and 250 mg for regimen B.

In the 14 objective responders in the high dose group, there were only 4 patients with a previous response to endocrine treatment. In the low dose group, only two of the 6 responders had responded to prior hormone therapy.

After CMF was started, 2 patients, both in the high dose group, achieved an improved response category, complete in one instance.

### *Toxicity*

The toxic manifestations are listed in Table III. The tolerance to cytotoxic chemotherapy was generally acceptable and overlapping in both groups of patients for nausea, vomiting, alopecia and myelosuppression. There were no cases of thrombocytopenia in either arm of the trial. In 18 patients on regimen A and 19 on regimen B, a delay of one week in the administration of adriamycin was necessary on one or more occasions. In three patients on regimen A and two in regimen B, a white blood cell count of $<2,000\,\mu l^{-1}$ necessitated a delay in treatment of two weeks before treatment could be resumed. In one patient on regimen A there was prolonged leucopenia and chemotherapy had to be discontinued. There was no deterioration in left ventricular ejection fraction assessed by isotopic gated angiocardiography nor congestive heart failure in any of the patients studied. One case of septicaemia occurred and was treated successfully with antibiotics, but this was while on treatment with CMF. No drug related deaths occurred. The median survival of non-responders on each regimen was similar (regimen A – 10 months, regimen B – 8 months) indicating that there was no additional toxicity of high dose treatment in non-responders.

**Table III** Toxicity

| | *Number of patients* | |
|---|---|---|
| | *Regimen A (n=24)* | *Regimen B (n=24)* |
| Haematological toxicity | | |
| Leukocyte nadir ($\mu l^{-1}$)[a] | | |
| ≧4,000 | 5 | 6 |
| 2,000–3,999 | 15 | 16 |
| 1,000–1,999 | 4 | 2 |
| Platelet nadir ($\mu l^{-1}$)[a] | | |
| <100.000 | 0 | 0 |
| Nausea and vomiting[b] | 24 | 24 |
| Alopecia | 24 | 22 |
| Cardiotoxicity | 0 | 0 |
| Septicaemia[c] | 1 | 0 |

[a]At day 21 of course; [b]WHO grade 2 or 3; grade 4 not observed; and [c]occurred during subsequent CMF.

### Discussion

The results of this prospective, randomised trial show a significantly better therapeutic effect for a high dose of adriamycin ($70\,mg\,m^{-2}$ i.v. 3-weekly) compared to a low dose regimen ($35\,mg\,m^{-2}$) in patients with disseminated breast carcinoma. The response rates were 58% and 25% respectively and the median survival was 20 months for the high dose and 8 months for the low dose group. Because of the marked and significant difference in overall response rate between the two regimens after the accrual of 48 patients, it was deemed unethical to continue further entry to the trial.

The response rate of 58% with the high dose schedule of adriamycin $70\,mg\,m^{-2}$ i.v. 3-weekly for 8 cycles is identical to previous experience with adriamycin either alone or in combination (Steiner *et al.*, 1983; Amiel *et al.*, 1985). No potential clinical advantage was demonstrated for using a low dose of adriamycin to enable the duration of drug administration to be doubled. The results here confirm previous findings indicating that an objective response to previous endocrine treatment does not predict response to subsequent cytotoxic chemotherapy (Creech *et al.*, 1980). The administration of adriamycin in either regimen was not associated with cardiotoxicity. For both regimens, there was an acceptable tolerance compatible with out-patient care.

In conclusion, the present prospective randomised clinical trial demonstrates that used at 3-weekly intervals there is a significant advantage for higher rather than lower doses of adriamycin in the treatment of patients with disseminated breast carcinoma. It is possible that lower doses given at more frequent intervals could give results equivalent to high dose treatment, but probably at greater inconvenience to the patient. Because treatment for advanced breast cancer is given to relieve symptomatic disease and because palliation results from achieving objective responses, the result of this trial is of some importance for guiding the optimal selection of treatment for this disease.

### References

AMIEL, S.A., STEWART, J. F., EARL, H.M., KNIGHT, R.K. & RUBENS, R.D. (1985). Adriamycin and Mitomycin C as initial chemotherapy for advanced breast cancer. *Eur. J. Cancer Clin. Oncol.*, **21**, 1475.

CHLEBOWSKY, R.T., PUGH, R., WEINER, J.M., BLOCK, J.B. & BATEMAN, J.R. (1983). Doxorubicin and CCNU with or without vincristine in patients with advanced refractory breast cancer. A randomized trial. *Cancer*, **52**, 606.

CREECH, R.H., CATALANO, R.B. & SHAH, M.K. (1980). An effective low dose adriamycin regimen as secondary chemotherapy for metastatic breast cancer patients. *Cancer*, **46**, 433.

FREI, E. & CANELLOS, G.P. (1980). Dose: A critical factor in cancer chemotherapy. *Am. J. Med.*, **69**, 585.

HAYWARD, J.L., RUBENS, R.D., CARBONE, P.P., HEUSON, J-C., KUMAOKA, S. & SEGALOFF, A. (1977). Assessment of response to therapy in advanced breast cancer. *Br. J. Cancer*, **35**, 292.

O'BRYAN, R.M., BAKER, L.H., GOTTLEIB, J.E. & **0** others (1977). Dose response evaluation of adriamycin in human neoplasia. *Cancer*, **39**, 1940.

STEINER, R., STEWART, J.F., CANTWELL, B.J.M., MINTON, M.J., KNIGHT, R.K. & RUBENS, R.D. (1983). Adriamycin alone or combined with vincristine in the treatment of advanced breast cancer. *Eur. J. Cancer Clin. Oncol.*, **19**, 1553.